# *N*‐Glycosylation Influences the Heterologous Expression of an Unspecific Peroxygenase From *Marasmius rotula* in *Saccharomyces cerevisiae*

**DOI:** 10.1111/1751-7915.70418

**Published:** 2026-07-19

**Authors:** Marina Schramm, Kai‐Uwe Schmidtke, Yvonne Kolaczek, Nico Jehmlich, Martin Hofrichter, Katrin Scheibner

**Affiliations:** ^1^ Institute of Biotechnology Brandenburg University of Technology Cottbus‐Senftenberg Senftenberg Germany; ^2^ Department of Molecular Toxicology Helmholtz‐Centre for Environmental Research, UFZ Leipzig Germany; ^3^ International Institute Zittau Dresden University of Technology Zittau Germany

## Abstract

*N*‐Linked glycosylation can have a significant impact on the yield of heterologously expressed proteins secreted by *Saccharomyces cerevisiae* . The yeast is a widely used host for the expression of unspecific peroxygenases (UPOs), a subclass of peroxide‐dependent oxidoreductases with high potential for industrial biocatalytic applications. However, the effects of *N*‐glycosylation on the expression of recombinant UPOs have not yet been investigated. Here, we studied respective protein modification on the expression of a UPO from *Marasmius rotula*, belonging to the protein subfamily of short peroxygenases, in *S. cerevisiae* . Two of the three *N*‐glycosylation sites that are actually occupied in r*Mro*UPO were eliminated by substituting asparagine at positions N43 and N151 with serine. The single substitutions led to reduced amounts of secreted r*Mro*UPO, with N43S having the highest impact (almost four times lower protein amount compared to the native enzyme) and N151S having a moderate effect. In the next step, the glycosylation probability at position 43 was enhanced by replacing the serine in the corresponding sequon with threonine, which increased the expression of the r*Mro*UPO variant. The concentration of active UPO in the concentrated culture supernatant of the glycosylation‐optimised variant S45T was twice as high as that of the native r*Mro*UPO. The results suggest that *N*‐linked glycosylation is an important factor for successful heterologous UPO expression in *S. cerevisiae* .

## Graphical abstract

*N*‐Linked glycosylation is evidently an important factor for the successful heterologous expression of unspecific peroxygenases (UPOs) in *Saccharomyces cerevisiae* , as demonstrated using a short UPO from *Marasmius rotula*. By increasing the probability of glycosylation within a sequon, the yield of extracellular UPO can be substantially increased.

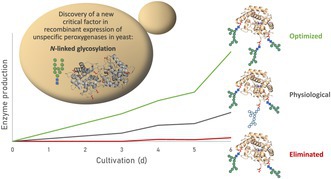

## 1 Introduction

Biocatalysis offers sustainable and efficient routes for the synthesis of pharmaceuticals, agrochemicals and fine chemicals. The industrial scalability of biocatalytic processes, however, depends on the broad availability of robust enzymes. Unspecific peroxygenases (UPOs; EC 1.11.2.1), secreted by fungi, are particularly promising biocatalysts due to their ability to transfer oxygen from H_2_O_2_ to both activated and non‐activated hydrocarbons, enabling challenging oxyfunctionalisation reactions such as hydroxylation and epoxidation that are difficult to achieve via conventional chemistry (Wang et al. 2017; Kiebist et al. 2019; Hofrichter et al. 2022; Kinner et al. 2021). As extracellular, glycosylated enzymes, UPOs are relatively stable and self‐sufficient, requiring only H_2_O_2_ as co‐substrate for activation and small amounts of Mg^2+^ for heme stabilisation, with no need for auxiliary electron transport proteins. Their catalytic versatility encompasses P450‐like monooxygenase and peroxidase‐like activities, making them ideal for the synthesis of specialty chemicals (Hofrichter et al. 2015, 2020). Thus, UPOs have been shown to form a variety of pharmaceutical derivatives mimicking human cytochrome P450 metabolites, including those of volixibat (Kiebist et al. 2015), cyclophosphamide (Steinbrecht et al. 2020), propranolol (Gomez De Santos et al. 2018), diclofenac (Kinne et al. 2009), testosterone (Kiebist et al. 2017), corticosteroids (Ullrich et al. 2018), polyunsaturated fatty acids (König et al. 2022) and clopidogrel (Kiebist et al. 2021).

UPOs investigated so far show individual substrate spectra, highlighting the enormous biocatalytic potential of the several thousand putative UPO genes that can be found in sequence databases (Hofrichter et al. 2022). In order to gain access to this huge pool of biotechnologically relevant enzymes, successful attempts have been made to express them heterologously in established yeast hosts such as *Saccharomyces cerevisiae* and *Pichia pastoris* (syn. *Komagataella phaffii*) (Molina‐Espeja et al. 2014, 2015; Gomez De Santos et al. 2021; Püllmann and Weissenborn 2021; Püllmann et al. 2021; Rotilio et al. 2021; Bormann et al. 2022; Ebner et al. 2023; Besleaga et al. 2024; Fu et al. 2024; Sánchez‐Moreno et al. 2024). Although both of these organisms lack a putative UPO gene (Hofrichter et al. 2022) in their genomes, they are capable of producing large amounts of UPOs in some cases. The highest value reported so far is 1.18 g L^−1^ for a recombinant UPO from *Marasmius fiardii* (r*Mfi*UPO) expressed in *P. pastoris* (Fu et al. 2024). However, there is no guarantee that every UPO gene is actually expressed in yeast, and if so, the amounts produced are often low; the reasons for this are largely unknown.

The success of functional gene expression in a foreign host is influenced at very different levels of protein synthesis. Almost all attempts to successfully express UPOs heterologously initially focused on optimising the processes at the DNA and/or RNA level. It is common practice to use codons that are particularly suitable for yeasts when designing the reverse‐translated gene (codon‐optimised sequences). Appropriate transcriptional and translational regulatory elements as well as the copy number of the expression cassette also have a major influence on heterologous UPO production (Püllmann and Weissenborn 2021; Besleaga et al. 2024; Zhao et al. 2024). At the protein level, optimisation often focuses on the identification of signal peptides that trigger active secretion, are recognised as such by the host protein synthesis machinery and interact well with the mature protein (Püllmann et al. 2021; Camboni et al. 2025).

If the protein is to be secreted, a further influencing factor often comes into play: glycosylation. *N*‐Linked glycosylation is an important co‐ and post‐translational modification, in which one or more sugar molecules (glycan moiety) are bound to a nitrogen atom in the side chain (amide group) of an asparagine residue within a protein (Aebi 2013). *N*‐Glycosylation occurs in the endoplasmic reticulum (ER) and is guided by a specific sequence motif, the sequon: ‐Asn‐X‐Ser/Thr‐ (where X is any amino acid except proline). The glycan moiety is pre‐assembled on a lipid carrier (dolichol) and then transferred en bloc to the nascent protein by a membrane‐bound oligosaccharyltransferase (OST, EC 2.4.99.18). After transfer, the glycan undergoes further trimming and remodelling in the ER and Golgi apparatus, influencing protein folding, stability and function (Skropeta 2009; Roth et al. 2010).

*N*‐Glycosylation in *S. cerevisiae* differs from that in most other fungal organisms, particularly with regard to the extent and structure of the glycan moieties. Thus, the yeast is notorious for forming extremely long outer chains consisting of 50–150 mannose units, a process known as hyper‐glycosylation of the high‐mannose type. This leads to large, heterogeneous and immunogenic glycans on corresponding proteins, which are often unsuitable for therapeutic applications (Laukens et al. 2015). Compared to *S. cerevisiae* , *P. pastoris* is advantageous in terms of glycosylation, as it attaches fewer mannose residues to proteins, although it is still prone to over‐glycosylation (Vieira Gomes et al. 2018). Given this context, it therefore seems plausible that fungal UPOs heterologously expressed in *S. cerevisiae* (or *P. pastoris*) do not undergo the same glycosylation as in homologous formation, which may lead to defective protein structure and/or impaired protein function (i.e., low enzymatic activities).

Glycosylation has not yet been further studied with regard to its influence on the heterologous expression of UPOs in yeast. We therefore investigated the effect of altered *N*‐glycosylation sites on the expression level of a recombinant UPO from *Marasmius rotula* (r*Mro*UPO) in *S. cerevisiae* .

## 2 Material and Methods

All chemicals were purchased from Sigma‐Aldrich, Schnelldorf, Germany, and were of reagent‐grade purity unless otherwise stated. The most important methods are described below; additional methods, along with their corresponding references, are listed in the Supporting Information.

### 2.1 In Silico Analyses

The in silico nucleic acid sequence work was carried out using SnapGene (version 8.0, SnapGene software; www.snapgene.com). The signal peptide cleavage site between amino acid 20 and 21 in the *Mro*UPO gene was identified using SignalP6.0 (Teufel et al. 2022). Putative *N*‐glycosylation sites in the primary sequence of the mature *Mro*UPO were identified with NetNGlyc—1.0, using default parameter settings (Gupta and Brunak 2002). Visualisation of the protein model of *Mro*UPO (PDB# 5FUJ) and its variants was done with PyMOL (version 2.5.2, Schrödinger LLC).

### 2.2 Cloning

For expression in yeast, the protein sequence of *Mro*UPO (with its native signal peptide; see (Sánchez‐Moreno et al. 2024)) was reverse‐translated using the optimal codons for *S. cerevisiae* using the SnapGene software. The gene was synthesised by GeneArt (Thermo Fisher Scientific, Germany) and cloned into a pYES2 vector (Thermo Fisher Scientific, Germany) that has an additional, reportedly stronger, terminator region of the *S. cerevisiae* *DIT1* gene (Yamanishi et al. 2013) downstream of the gene of interest. Mutants of *Mro*UPO were generated by site‐directed mutagenesis via PCR using appropriate primers. The *Escherichia coli* strain NEB10‐*beta* was used for transformations and plasmid propagation. The sequences of the inserts in the plasmids were analysed by Sanger sequencing; clones with correct inserts were additionally sequenced across the entire plasmid with Nanopore Sequencing Technology. All sequencing work was done by Eurofins Genomics (Cologne, Germany).

### 2.3 Enzyme Production

*S. cerevisiae* INVSc1 was freshly transformed with plasmids containing the *Mro*UPO gene or its mutants. For enzyme production, a colony of each transformed yeast clone was used for inoculating 500‐mL flasks containing 100 mL SC minimal medium (5 g L^−1^ ammonium sulphate, 1.7 g L^−1^ YNB w/o amino acids and w/o ammonium sulphate, 1.92 g L^−1^ yeast synthetic dropout w/o uracil) with 20 g L^−1^ glucose. The flasks were incubated at 30°C and 160 rpm for 2 days. 50 mL of these precultures were transferred to 500‐mL flasks, containing 100 mL expression medium (30 g L^−1^ galactose, 5 g L^−1^ ammonium sulphate, 1.7 g L^−1^ YNB w/o amino acids and w/o ammonium sulphate, 1.92 g L^−1^ yeast synthetic dropout w/o uracil, 140 mM potassium phosphate buffer pH 6.0, 1.17 g L^−1^ MgSO_4_, 0.42 g L^−1^ FeSO_4_, 3% ethanol, 25 mg L^−1^ chloramphenicol). The cultures were incubated at 24°C and 160 rpm for 6 days. Supernatants were separated from the cells by centrifugation at 5000 *g* for 20 min, and respective samples were pooled and concentrated using a Minimate TFF system (Pall Corporation, New York, USA) with a 10‐kDa cut‐off membrane. Further concentration was accomplished with VivaSpin Turbo 15 with a 10‐kDa cut‐off membrane (Sartorius AG, Göttingen, Germany). Overall, the supernatants were concentrated 150‐fold.

The major isoenzyme of the wild‐type UPO from the fungus *Marasmius rotula* (wt*Mro*UPO), which served as a template for the recombinant enzyme, was used as the reference protein and homologously produced as described by Gröbe et al. (2011). Modifications to the enzyme purification procedure are described in detail in the Supporting Information.

### 2.4 Enzyme Characterisation

#### 2.4.1 *N*‐Glycosylation Status

*N*‐glycosylated sites in wt*Mro*UPO and r*Mro*UPO were identified by PNGase F digestion of the proteins and following mass spectrometric analysis. Deglycosylation of the enzyme samples with PNGase F (New England Biolabs GmbH, Frankfurt am Main, Germany) was done under denaturing conditions according to the manufacturer's instructions. The deglycosylated protein samples were separated on a protein gel (see below) and the appropriate bands were cut out of the gel. The following digestion with trypsin and analysis by mass spectrometry are described in detail in the Supporting Information.

#### 2.4.2 SDS‐PAGE

Protein concentration in the samples was determined by measuring the absorbance at 280 nm, where 1 Abs_280_ corresponds to approximately 1 mg mL^−1^ protein. Protein samples (0.8 μg for wt*Mro*UPO and 7 μg for all other samples) were mixed with Laemmli sample buffer and 50 mM dithiothreitol to establish reducing conditions (or without this compound for normal non‐reducing conditions), heated at 95°C for 5 min and separated on Bolt 10% SDS‐PAGE using a mini gel tank (Life Technologies, Carlsbad, CA, USA). The pre‐stained ProSieve QuadColor Protein Marker (Lonza, Basel, Switzerland) was used as a reference. The gels were stained with Quick Coomassie (SERVA Electrophoresis GmbH, Heidelberg, Germany). Gel analyses were performed using the VWR Gel Doc Software (version 4.3.17.0; VWR International, Leuven, Belgium).

#### 2.4.3 UPO Concentration

The concentration of active wt*Mro*UPO as well as r*Mro*UPO and its variants was estimated using the corresponding carbon monoxide difference spectra (Otey 2003). The spectrum was recorded between 400 and 500 nm with a microplate reader (CLARIOstar Plus, BMG LABTECH GmbH, Germany). Then, the microtiter plate with the reduced samples was flushed with carbon monoxide (CO) and the spectrum was recorded again. To calculate the enzyme concentrations, an extinction coefficient of ε_444–490_ = 107 mM^−1^ cm^−1^ was used, as described for the recombinant UPO variant PaDa‐I (Tieves et al. 2019).

#### 2.4.4 Enzyme Activity Assay

Peroxygenase activity was measured by following the oxidation of veratryl alcohol into veratraldehyde (ε_310_: 9300 M^−1^ cm^−1^) in McIlvaine buffer at pH 5.5 (Ullrich et al. 2004) using a BioMate 160 UV/VIS photometer (Thermo Fisher Scientific, Germany). Reactions were started by addition of 2 mM H_2_O_2_.

## 3 Results and Discussion

### 3.1 *N*‐Glycosylation Sites in *Mro*UPO

There are several studies demonstrating an impact of *N*‐glycosylation on the expression and activity of different recombinant proteins heterologously produced in yeast. Some of these were summarised in a review from 2018 (Ge et al. 2018). While a number of studies have shown that (partial) deletion of native *N*‐glycosylation sites in a glycoprotein can lead to reduced secretion levels (Turner et al. 2006; Han et al. 2014; Wang et al. 2020), other studies have reported opposite results (Wang et al. 2000; Hoshida et al. 2013). Since studies on the effect of *N*‐glycosylation on the secretion of recombinant glycoproteins in *P. pastoris* or *S. cerevisiae* have been contradictory and, moreover, protein‐specific, it was not possible to predict what effects *N*‐glycosylation would have on the heterologous expression of UPOs. Therefore, the influence of *N*‐glycosylation on the expression of a short UPO from the basidiomycetous fungus *Marasmius rotula* in *S. cerevisiae* was investigated.

**FIGURE 1:**
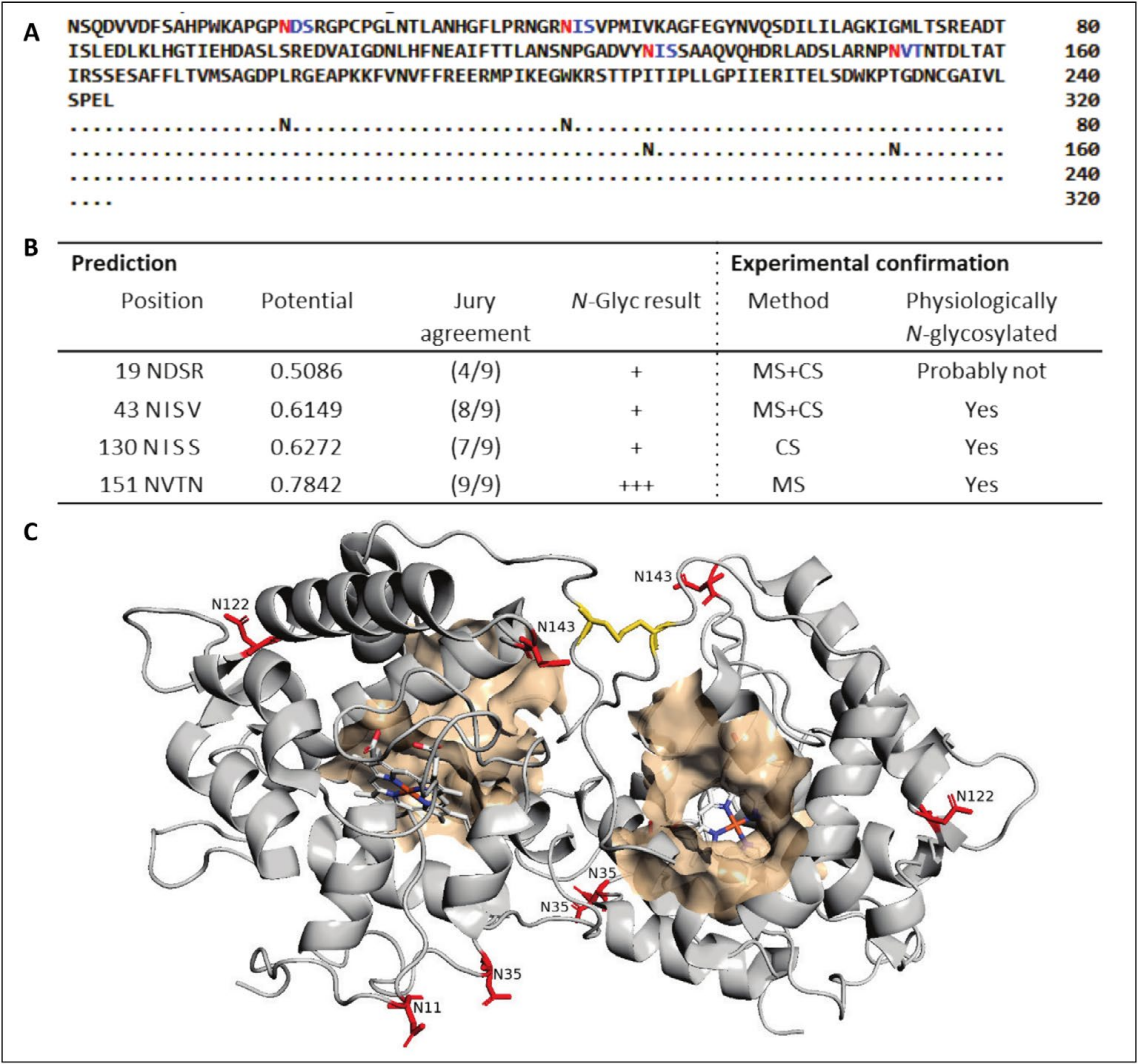
*N*‐glycosylation sites in *Mro*UPO. (A) Sequence of the mature *Mro*UPO with highlighted canonical sequons (N‐X‐S/T). (B) Predicted and experimentally confirmed *N*‐glycosylated sites in wt*Mro*UPO. For the prediction and evaluation of the probability, the tool NetNGlyc—1.0 was used. The experimental confirmation was done by mass spectrometry (MS) analysis of PNGase F‐treated wt*Mro*UPO. Additional information was obtained from publicly available crystal structures (CS) of wt*Mro*UPO (PDB# 5FUJ/5FUK). Further details can be found in the text. (C) Visualisation of the potential *N*‐glycosylation sites within the protein model of the homodimeric wt*Mro*UPO (PDB# 5FUJ). The ribbon model is shown as the physiological homodimer, with the intermolecular disulfide bond highlighted in yellow and the eight (2 × 4) potentially glycosylated Asn residues marked in red. For better visual clarity, the heme access channels are highlighted in orange.

This homodimeric UPO can be homologously produced in high yields of up to 445 mg L^−1^ using the wild‐type fungus (Gröbe et al. 2011). The protein sequence of the mature *Mro*UPO was analysed with an online tool (NetNGlyc) designed for the prediction of *N*‐linked glycosylation sites in human proteins (Gupta and Brunak 2002). Four potential glycosylation sites coded by canonical Asn‐Xaa‐Ser/Thr‐sequon sequences were identified within the *Mro*UPO protein, but with differing probabilities for glycosylation (Figure 1A,B). The Asn at position 151 has the highest probability of being glycosylated because the corresponding sequon contains Thr, which is favoured over Ser in terms of glycosylation efficiency (Breuer et al. 2001). All Asn residues, with the exception of N130, are freely accessible within loop regions (Figure 1C). N130 is located at the start of an α‐helix. The asparagine at position 151 in each monomer faces the entrance to the heme access channel of the other monomer.

We examined the actual *N*‐glycosylation status of *Mro*UPO expressed recombinantly in *S. cerevisiae* (r*Mro*UPO) and in the wild‐type fungus *Marasmius rotula* (wt*Mro*UPO) as reference (Figure 1B). Deglycosylation of the glycoproteins with PNGase F led to the deamidation of asparagine residues to which an *N*‐acetylglucosamine was bound, as evidenced by a corresponding mass shift detected by mass spectrometry (Figure S1A–C). The MS data were filtered for peptides that contained the canonical sequons. One sequon (N130‐I‐S) could not be identified, likely because it is contained within the largest peptide resulting from trypsin digestion, which consists of 40 amino acids; for technical reasons, it was not possible to analyse this peptide properly. The other data clearly showed deamidation of N43 and N151 in both enzymes (Figure S2A,B), suggesting that *N*‐linked glycan moieties were attached at these positions.

The available crystal structures for wt*Mro*UPO (PDB# 5FUJ and 5FUK) confirmed the presence of oligosaccharide chains at position N43 (N35 in the models) and also at position N130 (N122 in the models) that could not be demonstrated by our MS analysis. However, N151 (N143 in the models) was not consistently glycosylated in these models. Carbohydrates are generally difficult to model because they are less ordered and exhibit a high degree of flexibility. They often do not crystallise properly and are therefore not recognised as such (Van Beusekom et al. 2019). The MS data regarding glycan attachment to the asparagine residue with the lowest predicted probability, N19, were inconclusive for both enzymes. Taking into account the information from the PDB, we assume that this site is not glycosylated.

**FIGURE 2:**
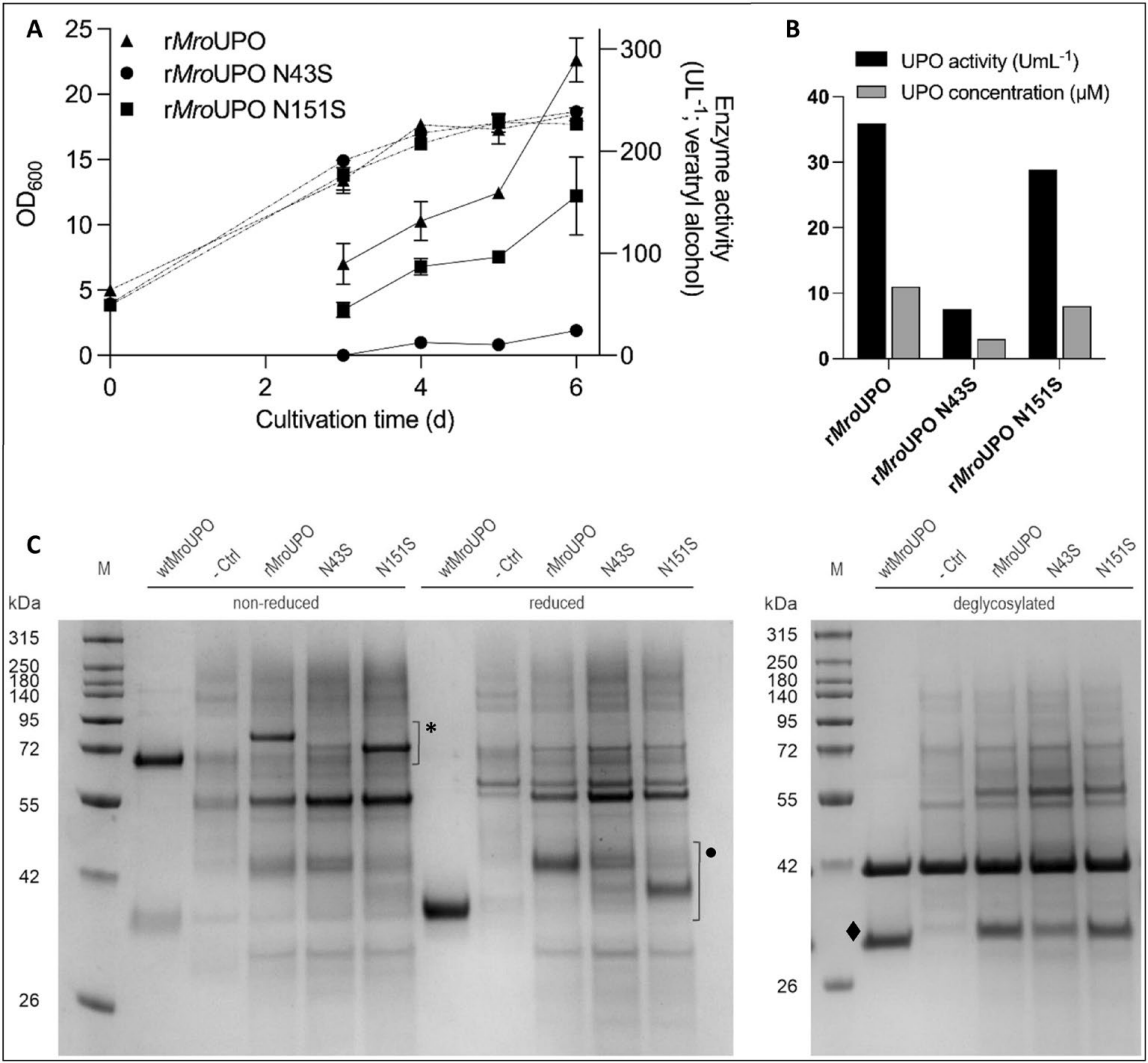
(A) Expression of r*Mro*UPO and its partially unglycosylated variants N43S and N151S in *S. cerevisiae* . OD_600_ and UPO activity were monitored spectrophotometrically over the cultivation time. Extracellular UPO activity was measured in the culture supernatants with veratryl alcohol as substrate (that is oxidised to veratraldehyde). Dashed lines represent the optical density at 600 nm (OD_600_, as a measure for cell density and hence yeast biomass) and the solid lines represent the time course of the UPO volume activity. The data points represent mean values of biological duplicates with standard deviation. (B) UPO activity and concentration in the concentrated culture supernatants; the former was determined by conversion of veratryl alcohol and the latter by measuring the CO‐difference spectra. (C) SDS‐PAGE of concentrated culture supernatants of *S. cerevisiae* containing native r*Mro*UPO or the partially unglycosylated variants N43S and N151S. The protein samples comprised the non‐reduced r*Mro*UPO (homo‐dimeric state), the reduced forms (monomeric state) or the reduced and deglycosylated forms. Protein samples (7 μg) from *S. cerevisiae* cultures were used along with 0.8 μg of the purified wild‐type protein (wt*Mro*UPO) as reference. The prominent band in the deglycosylated samples is PNGase F (36 kDa). “*”—glycosylated dimeric *Mro*UPO (and variants); “•”—glycosylated monomeric *Mro*UPO (and variants); “♦”—deglycosylated monomeric *Mro*UPO (and variants); “‐Ctrl”—control yeast culture that expressed no UPO.

When we expressed *Mro*UPO heterologously in *S. cerevisiae* , the yield was very low compared to the wild‐type. Although MS analysis suggests that both enzymes probably have the same number of *N*‐glycosylated sites, the recombinant variant has an apparent molecular weight that is approximately 17% higher than that of wt*Mro*UPO (Figure 2C), which is likely due to a higher number of bound mannose units. However, the differences in glycosylation do not appear to have a significant effect on enzyme activity, as evidenced by the similar kinetic parameters for prototypical UPO substrates such as veratryl alcohol, 2,6‐dimethoxyphenol and 5‐nitrobenzodioxole (Table S1).

### 3.2 Effect of the Partial Absence of Glycosylation on the Expression of r*Mro*UPO

Since *N*‐glycosylation may influence expression and, consequently, the yield of secreted protein, we selected two of the confirmed *N*‐glycosylation sites to investigate how the partial absence of glycosylation at these positions affects the expression of r*Mro*UPO in *S. cerevisiae* . For a defunctionalisation experiment, a site closer to the N‐terminus (N43) and a site closer to the C‐terminus (N151) were selected. The Asn residues at these positions were substituted by Ser to obtain a nano‐environment that is as similar as possible in terms of structure, polarity and molecular volume (Grantham 1974). According to the computer models, the substitutions are unlikely to cause any significant changes in conformation, given the properties of the amino acids in question and their location on the protein's surface (Figure S3A,B‐1,B‐2).

r*Mro*UPO and its partially unglycosylated single mutants N43S and N151S were episomally expressed in *S. cerevisiae* under transcriptional control of the galactose‐inducible GAL1‐promoter. After six days of cultivation, supernatants containing the native r*Mro*UPO showed the highest UPO activity measured with veratryl alcohol as substrate (290 U L^−1^; Figure 2A). Cultures that produced the variant without glycosylation near the C‐terminus (N151S) exhibited an activity of 156 U L^−1^, which was approx. 46% lower than that of the original. The cultures with the N‐terminal variant N43S were even more severely affected by the lack of glycosylation and achieved only one tenth (25 U L^−1^) of the volume activity of the cultures with the native enzyme. According to the results of the optical density measurements (cell density ≈ biomass), the vitality of the cells was obviously not influenced by the expression of the different variants of the UPO gene (and can therefore be ruled out as an explanation for the differences in activity).

After concentrating the culture supernatants by ultrafiltration with a 10‐kDa cut off membrane, the activities measured with veratryl alcohol were 35.9 U mL^−1^ for r*Mro*UPO as well as 7.6 and 28.9 U mL^−1^ for the variant N43S and N151S, respectively (Figure 2B). As in the present case, when working with UPOs, there is sometimes a discrepancy between the expected activity values—based on measurements in the original, unprocessed culture supernatants—and the actual values following ultrafiltration. The activity often appears to increase after filtration. We assume that low‐molecular‐weight components in the supernatant either reversibly influence the activity or mask it by acting as a competitive substrate (Ullrich et al. 2009).

The proteins in the concentrated supernatants from *S. cerevisiae* cultures were analysed by gel electrophoresis with wt*Mro*UPO as reference (Figure 2C). Based on the protein sequence, the monomer of mature *Mro*UPO was calculated to have a molecular weight of 26 kDa. Including the mass contribution by glycosylation, the monomer of wt*Mro*UPO was reported to have an apparent molecular weight of 32 kDa according to SDS‐PAGE (Gröbe et al. 2011). As the protein marker used here was pre‐stained, there may be discrepancies in the determination of the molecular weight. Under non‐reducing conditions, both native r*Mro*UPO and the mutants exist in the homo‐dimeric state, which corresponds to the physiological form of wt*Mro*UPO (confirmed by the 5FUJ/5FUK crystal structures of the homologously expressed wild‐type enzyme, which were deposited in the Protein Data Bank/PDB in 2016 and also later reported in (Linde et al. 2022)). The dimer is formed by a disulfide bond between the C‐terminal cysteines. Judging by the SDS‐PAGE results, it appears that some of the protein is present as a monomer even under non‐reducing conditions. In this context, this appears to be more pronounced in r*Mro*UPO than in the wild‐type and the variant N151S. However, the exact dimer‐to‐monomer ratio could not be determined here. No conclusion could be drawn regarding the variant N43S because the amount of the target protein was too low.

The glycosylated recombinant *Mro*UPO produced in *S. cerevisiae* was found to have a higher apparent molecular weight than the wild‐type enzyme, which may be attributable to a different glycan pattern. The elimination of one glycosylation site in variant N43S and N151S resulted in modified proteins exhibiting different mobility in the gel compared to native r*Mro*UPO. The lower number of bound sugar molecules reduced the molecular weight of the variants by approximately 8%. PNGase F‐treatment and following MS‐analysis of the mutants revealed that the remaining sequons were unchanged with respect to their glycosylation status (Figure S2C,D).

The amount of active UPOs in the concentrated culture supernatants was determined based on a widely used method for the specific detection of P450 enzymes, as well as UPOs, by measuring CO difference spectra (Guengerich et al. 2009; Tieves et al. 2019; Hilberath et al. 2022). The calculated concentrations were 11 μM for r*Mro*UPO, 3 μM for N43S and 8 μM for N151S (Figure 2B), corresponding to 1.9, 0.5 and 1.4 mg L^−1^ UPO protein in the original culture liquids, and specific activities of 126.0, 101.3 and 137.6 U mg^−1^, respectively. It is known that altered glycosylation can affect the specific activity of enzymes (Skropeta 2009). However, since the enzymes used here were not purified (which would have gone beyond the scope of this study) and, for example, the value for native r*Mro*UPO in the other experiment (see next section) was about 20% lower, the values determined appear to fall within the range of biological variation. It can therefore be reasonably assumed that, in this case, partial deglycosylation has no substantial effect on the specific activity. Overall, inactivation of the *N*‐glycosylation sites in r*Mro*UPO resulted in a 3.8‐fold and 1.4‐fold reduction, respectively, in the yield of secreted active enzyme, depending on whether the site was located at the N‐terminus or the C‐terminus.

The negative effects of removing *N*‐glycosylation sites were also observed for other secreted recombinant proteins expressed in yeast, where secretion decreased or stopped completely (Turner et al. 2006; Han et al. 2014; Wang et al. 2020). One explanation could be an impaired folding process, since lectin chaperones such as calnexin recognise specific *N*‐glycans on newly synthesised glycoproteins in the ER and facilitate their folding (Roth et al. 2010). Proteins that are not folded correctly in the ER are eliminated by the endoplasmic reticulum‐associated protein degradation (ERAD) pathway (Roth et al. 2010). On the other hand, it is known that glycosylation sterically protects sensitive sites such as joints and linkers in the protein from attack by proteases (Rocamora et al. 2023). Therefore, it is also conceivable that the variants with reduced glycosylation were partially proteolytically degraded in the culture liquid.

### 3.3 Effect of an Improved Glycosylation Probability on the Expression of r*Mro*UPO

After observing a decrease in yield of extracellular r*Mro*UPO following the elimination of two individual glycosylation sites, we investigated the effects of the opposite approach, namely sequon optimisation. We focused on the sequon at position 43, which had a pronounced detrimental effect when defunctionalised, and attempted to enhance the probability for *N*‐glycosylation at this site. The co‐translational process of *N*‐glycosylation does not always proceed correctly. Under certain circumstances, sequons may be skipped by the OST (Shrimal et al. 2015), which can lead to incorrect processing of the protein molecule and its subsequent degradation. We therefore assumed that an increase in the probability of glycosylation would also increase the probability that correctly folded and processed r*Mro*UPO would be secreted into the medium. As mentioned above, Thr can trigger improved glycosylation in the Asn‐Xaa‐Ser/Thr sequons compared to serine (Bause 1983), as the extra β‐methyl group on threonine provides superior binding, conformational stability and increased catalytic efficiency for the OST (Gerber et al. 2013). NetNGlyc‐1.0 predicted an increase in the probability of *N*‐glycosylation at position N43 in the corresponding variant *Mro*UPO S45T from 0.6149 to 0.6791. The site is located at the surface of the protein and, according to the computer models, the substitution of Ser by Thr should not cause any significant changes in conformation (Figure S3C).

**FIGURE 3:**
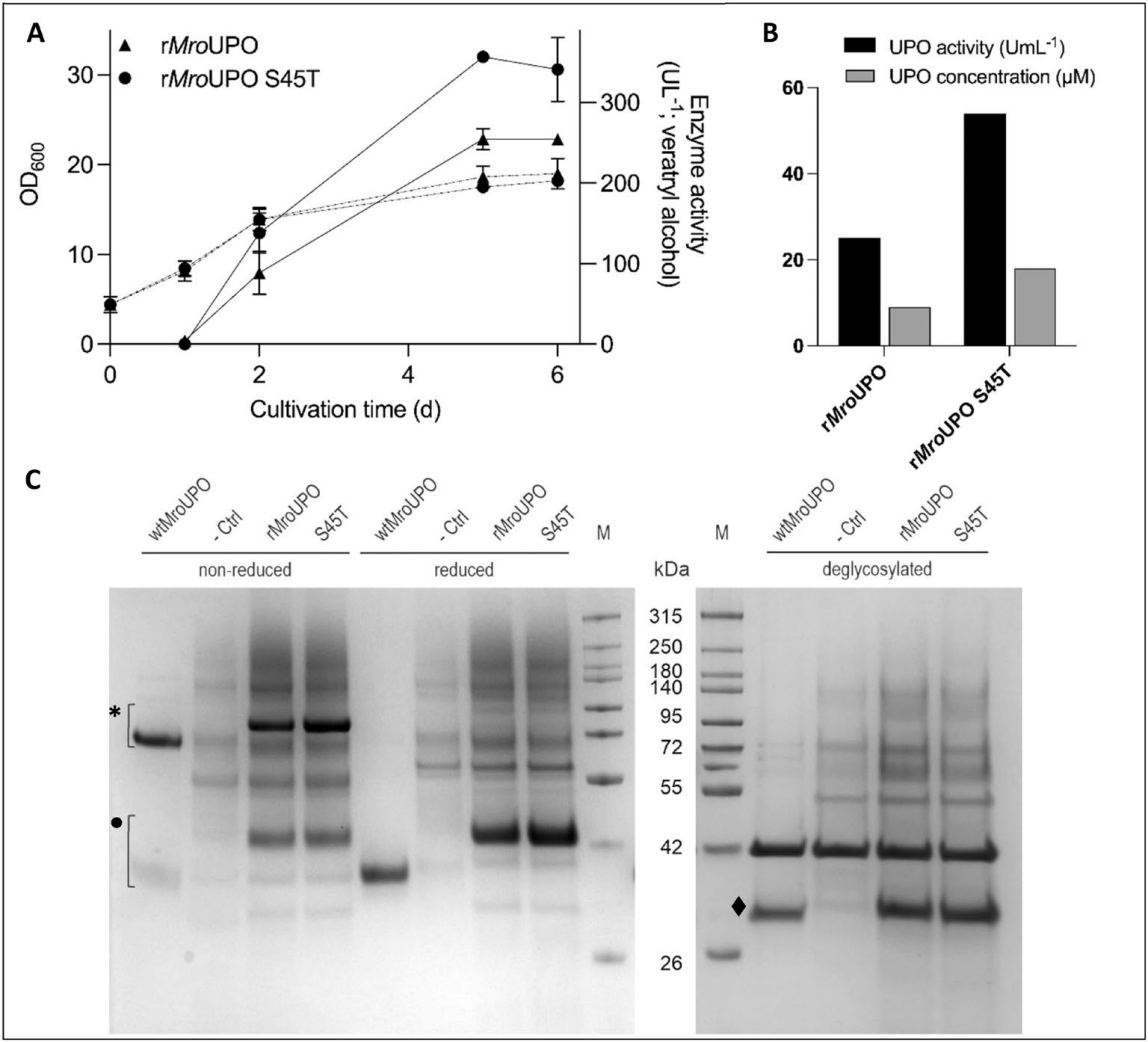
(A) Expression of native r*Mro*UPO and the glycosylation‐optimised variant S45T in *S. cerevisiae* . OD_600_ and UPO activity were monitored spectrophotometrically over the cultivation time. Extracellular UPO activity was measured in the culture supernatants with veratryl alcohol as substrate. The dashed lines correspond to the optical density at 600 nm (OD_600_, as a measure for cell density and hence yeast biomass), while the solid lines show the time course of UPO volume activity. The data points represent mean values of biological duplicates with standard deviation. (B) UPO activity and UPO concentration determined in the concentrated supernatants by veratryl alcohol oxidation and measuring the CO‐difference spectra, respectively. (C) SDS‐PAGE of concentrated culture supernatants of *S. cerevisiae* containing r*Mro*UPO and the glycosylation‐optimised variant S45T. The protein samples comprised the non‐reduced r*Mro*UPOs (homo‐dimeric state), the reduced forms (monomeric state) or the reduced and deglycosylated forms. Protein samples (7 μg) from *S. cerevisiae* cultures were used along with 0.8 μg of the purified wild‐type protein (wt*Mro*UPO) as reference. The prominent band in the deglycosylated samples is PNGase F (36 kDa). “*”—glycosylated dimeric *Mro*UPO (and the S45T variant); “•”—glycosylated monomeric *Mro*UPO (and the variant); “♦”—deglycosylated monomeric *Mro*UPO (and the variant); “‐Ctrl”—control yeast culture that expressed no UPO.

As before, native r*Mro*UPO and the variant r*Mro*UPO S45T were heterologously expressed in *S. cerevisiae* and the extracellular UPO activity was determined. On the fifth day of cultivation, the volume activity in the cultures of the glycosylation‐optimised mutant was almost 40% higher than in cultures with the native r*Mro*UPO (356 U L^−1^ compared to 254 U L^−1^, measured with veratryl alcohol as substrate; Figure 3A). All cultures showed a similar OD_600_ during cultivation, indicating that there were no significant differences in biomass formation due to possible metabolic impairment caused by the expression of the foreign genes.

After concentrating the culture supernatants by ultrafiltration with a 10‐kDa cut off membrane, the measured activities with veratryl alcohol were 25.2 U mL^−1^ for r*Mro*UPO and 54.0 U mL^−1^ for the variant S45T (Figure 3B). The higher UPO activity in the cultures with the glycosylation‐optimised variant was accompanied by a higher amount of UPO protein, as indicated by the measured concentrations and the intensity of the corresponding bands in the SDS‐PAGE (Figure 3B,C).

Since the number of sequons in r*Mro*UPO and the optimised variant is the same, no change in the degree of glycosylation was expected. This was in fact confirmed by the enzymes' nearly identical apparent molecular weights in the gel. In addition, PNGase F‐treatment and subsequent MS‐analysis of the mutant showed that the glycosylation status remained unchanged compared with r*Mro*UPO (Figure S2C,D). The ratio of dimer to monomer in the non‐reduced samples was similar as well. In this experiment, the concentration of active native r*Mro*UPO and of the variant r*Mro*UPO S45T in the concentrated supernatants was calculated to be 9 and 18 μM, respectively, according to the CO‐difference spectral assay. This corresponds to 1.6 and 3.2 mg L^−1^ UPO protein in the original (untreated) culture supernatants (at specific activities of 105.0 and 112.5 U mg^−1^), and means that the total amount of active, secreted UPO protein in cultures of the glycosylation‐optimised variant was twice as high as the amount of native r*Mro*UPO.

Optimisation of the probability for *N*‐glycosylation was also beneficial in case of the peptidase elastase where the substitution of Ser by Thr in the ‐Asn‐Xaa‐Ser‐ sequon resulted in a variant with 34% increased enzyme production (Han et al. 2015). Furthermore, the introduction of an additional *N*‐glycosylation sequon worked well with regard to the secretion of cutinase and antibody protein fragments in *S. cerevisiae* and *P. pastoris* (Sagt et al. 2000). That work also showed that the position of the introduced glycosylation site in the peptide chain was significant, as the closer it was to the N‐terminus, the greater the improvement in secretion. The presence of an *N*‐glycosylation sequon in the N‐terminal region could also explain the findings of a study, in which a shuffling library of three UPOs was examined, including a yeast‐optimised variant from *Agrocybe* (syn. *Cyclocybe*) *aegerita* (PaDa‐I), one from *Galerina marginata* and one from *Coprinopsis cinerea* (like *M. rotula*, the three fungal species belong to the filamentous agaric Basidiomycota) (Knorrscheidt et al. 2020). The five only active UPO chimeras that were generated all had exclusively the N‐terminal subunit of the well adapted PaDa‐I mutant. In the sequence of PaDa‐I, there is a sequon (Asn‐Ser‐Ser) in N‐terminal direction from the UPO‐specific heme‐binding PCP‐motif that is glycosylated (5OY1 crystal structure (Ramirez‐Escudero and Sanz‐Aparicio 2018)), and for which there is no equivalent in the sequences of the other two fungal enzymes.

There is still a lot of potential for further improvements in UPO expression using the “glycosylation approach”, since not only the sequons as such determine the glycosylation probability, but also the adjacent regions in the protein (Shakin‐Eshleman et al. 1996; Bañó‐Polo et al. 2011; Malaby and Kobertz 2013).

## 4 Conclusion

Proper *N*‐linked glycosylation is a process that can have a significant impact on the heterologous expression of proteins. This is obviously also true for the production of extracellular unspecific peroxygenases (UPOs) in yeast. Further studies are needed to clarify how and to what extent glycosylation and its localisation within the protein complex are, in fact, critical factors for the successful biosynthesis of recombinant UPOs and other heme‐thiolate proteins.

## Author Contributions

**Kai‐Uwe Schmidtke:** conceptualization, methodology, investigation, writing – review and editing. **Katrin Scheibner:** resources, supervision, funding acquisition, project administration. **Nico Jehmlich:** methodology, writing – review and editing. **Martin Hofrichter:** writing – review and editing, writing – original draft, resources. **Yvonne Kolaczek:** methodology, writing – review and editing. **Marina Schramm:** conceptualization, methodology, writing – original draft, writing – review and editing, investigation.

## Funding

This work was supported by Bundesministerium für Forschung, Technologie und Raumfahrt, 03LW0346.

## Conflicts of Interest

The authors declare no conflicts of interest.

## Supporting information


**Data S1:** mbt270418‐sup‐0001‐ExpData.zip.

**Figure S1:** Representative MS/MS spectra of the relevant peptides containing the canonical sequons (NXS/T) identified in the analysis of wt*Mro*UPO. (A) Identification of N5 in the potential *N*‐glycosylation site NDS in the peptide sequence APGPNDSRGPCPGLNTLANHGFLPR as a non‐deamidation site. Representative high‐resolution HCD‐MS/MS spectrum of the quadruply charged precursor ion assigned to non‐modified peptide sequence APGPNDSRGPCPGLNTLANHGFLPR, detected at 113.32 min. (B) Identification of N4 in NGRNISVPMIVK as a deglycosylation‐induced deamidation site. Representative high‐resolution HCD‐MS/MS spectrum of the triply charged precursor ion assigned to deamidated NGRNISVPMIVK, detected at 65.31 min. The peptide contains a potential *N*‐glycosylation site at Asn4 within the NIS sequon. The +0.984 Da mass shift is consistent with conversion of a formerly glycosylated Asn to Asp during deglycosylation. Fragment ions spanning N4 support localisation of the modification to this residue, while the complementary ion series confirms the peptide assignment. (C) Identification of N3 in the peptide sequence NPNVTNTDLTATIR as a deglycosylation‐induced deamidation site. Representative high‐resolution HCD‐MS/MS spectrum of the doubly charged precursor ion assigned to deamidated NPNVTNTDLTATIR, detected at 56.68 min. The peptide contains a potential *N*‐glycosylation site at Asn3 within the NVT sequon. The +0.984 Da mass shift is consistent with conversion of a formerly glycosylated Asn to Asp during deglycosylation. Fragment ions spanning N3 support localisation of the modification to this residue, while the complementary ion series confirms the peptide assignment. For alle MS/MS sectra, assigned b‐ and y‐type fragment ions are shown in red and blue, respectively, with neutral‐loss ions annotated where applicable. Unassigned peaks are shown in grey.
**Figure S2:** Analysis of the *N*‐glycosylation status of different variants of *Mro*UPO. The proteins were treated with PNGase F, digested with trypsin and analysed by mass spectrometry (MS). Asparagine residues in the potential sequons (N19‐D‐S; N43‐I‐S; N151‐V‐T) were analysed to determine whether they are deamidated (indicating prior binding of *N*‐acetylglucosamine) or not deamidated and therefore likely not glycosylated. Peptides with the respective sequons (deamidated or not deamidated) are shown as cumulated intensities for wtMroUPO and r*Mro*UPO (A) and the variants r*Mro*UPO N43S; N151S; S45T (C) or as a percentage (B and D). Peptides with the potential sequon N130‐I‐S were not found in the MS data.
**Figure S3:** Superimpositions of models of recombinant *Mro*UPO variants (dark grey) and wild‐type *Mro*UPO (light grey). The models were generated by homology modelling with PDB# 5FUJ as template using SWISS‐MODEL and aligned with PDB# 5FUJ in PyMOL. Relevant asparagine residues that were exchanged in the variants N43S (A) and N151S (B‐1 and B‐2) are shown as red sticks, the inserted serine is shown as blue sticks. (B‐2) shows the dimer at a different angle. Serine (S45) in the wt*Mro*UPO (green stick) was replaced by threonine (magenta stick) in the variant S45T (C). The RMSD between the aligned structures of N43S and wt*Mro*UPO, N151S and wt*Mro*UPO and S45T and wt*Mro*UPO was 0.065 Å, 0.066 Å and 0.076 Å.
**Table S1:** Kinetic parameters of wild‐type and recombinant *Mro*UPO. Ultrafiltrated culture supernatants as well as purified r*Mro*UPO and wt*Mro*UPO were used for their determination. The data obtained are in the same order of magnitude.

## Data Availability

The data presented in this study are available on request from the corresponding author.
