# Supplementary material for: N‐Glycosylation Influences the Heterologous Expression of an Unspecific Peroxygenase From Marasmius rotula in Saccharomyces cerevisiae
Source: Microb Biotechnol. 2026 Jul 19;19(7):e70418. doi: 10.1111/1751-7915.70418 (PMC13382365; doi:10.1111/1751-7915.70418)
Supplement: Supplementary file 2 — Figure S1: Representative MS/MS spectra of the relevant peptides containing the canonical sequons (NXS/T) identified in the analysis of wtMroUPO. (A) Identification of N5 in the potential N‐glycosylation site NDS in the peptide sequence APGPNDSRGPCPGLNTLANHGFLPR as a non‐deamidation site. Representative high‐resolution HCD‐MS/MS spectrum of the quadruply charged precursor ion assigned to non‐modified peptide sequence APGPNDSRGPCPGLNTLANHGFLPR, detected at 113.32 min. (B) Identification of N4 in NGRNISVPMIVK as a deglycosylation‐induced deamidation site. Representative high‐resolution HCD‐MS/MS spectrum of the triply charged precursor ion assigned to deamidated NGRNISVPMIVK, detected at 65.31 min. The peptide contains a potential N‐glycosylation site at Asn4 within the NIS sequon. The +0.984 Da mass shift is consistent with conversion of a formerly glycosylated Asn to Asp during deglycosylation. Fragment ions spanning N4 support localisation of the modification to this residue, while the complementary ion series confirms the peptide assignment. (C) Identification of N3 in the peptide sequence NPNVTNTDLTATIR as a deglycosylation‐induced deamidation site. Representative high‐resolution HCD‐MS/MS spectrum of the doubly charged precursor ion assigned to deamidated NPNVTNTDLTATIR, detected at 56.68 min. The peptide contains a potential N‐glycosylation site at Asn3 within the NVT sequon. The +0.984 Da mass shift is consistent with conversion of a formerly glycosylated Asn to Asp during deglycosylation. Fragment ions spanning N3 support localisation of the modification to this residue, while the complementary ion series confirms the peptide assignment. For alle MS/MS sectra, assigned b‐ and y‐type fragment ions are shown in red and blue, respectively, with neutral‐loss ions annotated where applicable. Unassigned peaks are shown in grey. Figure S2: Analysis of the N‐glycosylation status of different variants of MroUPO. The proteins were treated with PNGase F, digested with t [file MBT2-19-e70418-s002.docx]

***N*-Glycosylation influences the heterologous expression of an unspecific peroxygenase from *Marasmius rotula* in *Saccharomyces cerevisiae***

Marina Schramm, Kai-Uwe Schmidtke, Yvonne Kolaczek, Nico Jehmlich, Martin Hofrichter and Katrin Scheibner

**Supplementary information**

**Methods**

Proteomics

Each sample was prepared for proteolytic cleavage before mass-spectrometric analysis. The protein lysates were reduced (2.5 mM dithiothreitol for 1 h at 60 °C) and alkylated (10 mM iodoacetamide for 30 min at 37 °C). Proteolysis was performed overnight at 37°C using trypsin (Promega, Madison, WI, USA) with an enzyme: substrate ratio of 1:25. Extracted peptide lysate were desalted using a C18 ZipTip column (Merck Millipore), dissolved in 0.1% formic acid and injected into a nano liquid chromatography mass spectrometry system (nanoLC-MS/MS).

Mass spectrometry (MS) was performed using a Vanquish Neo nanoHPLC (Thermo Fisher Scientific) coupled to an Orbitrap Exploris™ 480 mass spectrometer (Thermo Fisher Scientific) operating in data-dependent acquisition (DDA) mode. Initial trapping occurred on a C18-reverse phase trapping column (Acclaim PepMapTM 100, 75 μm × 2 cm, particle size 3 μm, nanoViper, Thermo Fisher Scientific), followed by subsequent separation on a C18-reverse phase analytical column (Double nanoViper™ PepMap™ Neo, 75 μm × 150 mm, particle size 2 μm, Thermo Fisher Scientific). The separation utilized a two-step gradient employing mobile phases A (0.01% formic acid in H_2_O) and B (80% acetonitrile in H_2_O with 0.01% formic acid). During the first 65 minutes, the proportion of mobile phase B was increased from 4% to 30%, followed by a 30-min period, during which the proportion of mobile phase B rose from 30% to 55%. The flow rate was maintained at 300 nL per minute throughout the separation.

MaxQuant (Galaxy Version 2.0.3.0,(Pinter *et al.*, 2022)) was used for protein identification. For that, the measured MS/MS spectra (*.raw files) were searched against the common contaminant proteins and the protein sequences of *Mro*UPO, *Mro*UPO N43S, *Mro*UPO N151S, *Mro*UPO S45T. The following settings were chosen: enzyme specificity was trypsin with up to two missed cleavages allowed, a peptide ion tolerance of 10 ppm, and a 0.05 Da MS/MS tolerance. Oxidation (methionine), carbamidomethylation (cysteine) and deamidation (asparagine) were selected as a variable modification. Only peptides with a false discovery rate (FDR) 0.01 were considered as identified. To evaluate the *N*-glycosylation status, only peptides that contained the relevant sequon sequences (NDS; NIS/T; NVT) with a PEP <0.001 were considered. No peptides with the putative *N*-glycosylation site N130 were found, which was likely due to technical limitations, as this sequon is located in the largest fragment produced by trypsin digestion.

Purification of wt*Mro*UPO

The wildtype enzyme *Mro*UPO was produced and isolated as described previously (Gröbe *et al.*, 2011), but slightly modified. The culture filtrate was concentrated by two steps of ultrafiltration using two tangential-flow cassettes (Sartocon Slice Cassette, Hydrosart, cut-off 10 kDa, Sartorius, and Omega membrane, cut-off 10 kDa, Pall Life Sciences, Dreieich, Germany). All subsequent chromatographic purification steps were performed with an ÄktaFPLC™System (GE Healthcare Europe GmbH, Freiburg, Germany). First, the crude preparation was loaded onto a Q-Sepharose Fast Flow column (anion exchanger XK 26/20, Cytiva, Marlborough, MA, USA) and the proteins were eluted with a linear gradient of 0-0.7 M NaCl in 10 mM sodium acetate buffer (pH 6.0) at a flow rate of 8 mL min^-1^. The fractions containing UPO activity were pooled, concentrated and dialyzed against 10 mM sodium acetate (pH 4.7, 10 kDa cut-off Omega membrane, Pall Life Sciences). Subsequently, the samples were applied to a SP Sepharose™ Fast Flow column (cation exchange XK 26/20, Cytiva, Marlborough, MA, USA) and the proteins eluted with a linear gradient of 0-0.7 M NaCl in 10 mM sodium acetate buffer (pH 4.7) at a flow rate of 8 mL min^-1^. The *Mro*UPO-containing fractions were pooled, concentrated and dialyzed against 10 mM sodium acetate (pH 6, 10 kDa cut-off Omega membrane, Pall Life Sciences). The purity of the enzyme was determined spectrophotometrically based on the heme/protein ratio adapted to UPOs (*Reinheitszahl*, Rz_420 nm / 280 nm_) and was 1.6 (Theorell *et al.*, 1950).

Determination of kinetic parameters of wt*Mro*UPO and r*Mro*UPO

Kinetic parameters (k_cat_ – turnover number, K_m_ – Michaelis constant, k_cat_/K_m_ – catalytic efficiency) were determined for the prototypical UPO substrates veratryl alcohol (VA), 5-nitrobenzodioxole (NBD) and 2,6-dimethoxyphenol (DMP) by photometric assays using a plate reader. 180 µL of the reaction mixtures were added to 20 µL enzyme preparation to give the following final concentrations: 50 mM McIlvaine buffer pH 5.5 (pH 6.5 for NBD), varying concentrations (4-5,000 µM) of substrates (VA and DMP dissolved in H_2_O, NBD in acetonitrile), 2 mM H_2_O_2_ (1 mM for NBD) and 0.06 µM enzyme. The photometric measurements were started immediately after the reaction mixture was added to the enzyme preparation. The formation of veratraldehyde (ε_310_ = 9,300 M^−1^ cm^−1^), 5-nitrocatechol (ε_425_ = 9,700 M^−1^ cm^−1^) and coerulignone (ε_469_= 27,500 M^−1^ cm^−1^) was monitored using a CLARIOstar® Plus microplate reader (BMG LABTECH GmbH, Germany).

*In silico* analyses

Visualization of protein models of *Mro*UPO (PDB# 5FUJ) and variants was done with PyMOL (version 2.5.2, Schrödinger LLC). Homology modelling of the dimeric *Mro*UPO variants was carried out with the web-based tool SWISS-MODEL using the crystal structure PDB# 5fuj.1.A as template (Waterhouse *et al.*, 2018). The generated structures including that of wt*Mro*UPO (PDB# 5FUJ) were superimposed using the ‘align command’ in PyMOL.

**Results**

**A**

**B**

**C**

Figure S1: Representative MS/MS spectra of the relevant peptides containing the canonical sequons (NXS/T) identified in the analysis of wt*Mro*UPO. (A) Identification of N5 in the potential *N*-glycosylation site NDS in the peptide sequence APGPNDSRGPCPGLNTLANHGFLPR as a non-deamidation site. Representative high-resolution HCD-MS/MS spectrum of the quadruply charged precursor ion assigned to non-modified peptide sequence APGPNDSRGPCPGLNTLANHGFLPR, detected at 113.32 min. (B) Identification of N4 in NGRNISVPMIVK as a deglycosylation-induced deamidation site. Representative high-resolution HCD-MS/MS spectrum of the triply charged precursor ion assigned to deamidated NGRNISVPMIVK, detected at 65.31 min. The peptide contains a potential *N*-glycosylation site at Asn4 within the NIS sequon. The +0.984 Da mass shift is consistent with conversion of a formerly glycosylated Asn to Asp during deglycosylation. Fragment ions spanning N4 support localization of the modification to this residue, while the complementary ion series confirms the peptide assignment. (C) Identification of N3 in the peptide sequence NPNVTNTDLTATIR as a deglycosylation-induced deamidation site. Representative high-resolution HCD-MS/MS spectrum of the doubly charged precursor ion assigned to deamidated NPNVTNTDLTATIR, detected at 56.68 min. The peptide contains a potential *N*-glycosylation site at Asn3 within the NVT sequon. The +0.984 Da mass shift is consistent with conversion of a formerly glycosylated Asn to Asp during deglycosylation. Fragment ions spanning N3 support localization of the modification to this residue, while the complementary ion series confirms the peptide assignment. For alle MS/MS sectra, assigned b- and y-type fragment ions are shown in red and blue, respectively, with neutral-loss ions annotated where applicable. Unassigned peaks are shown in grey.


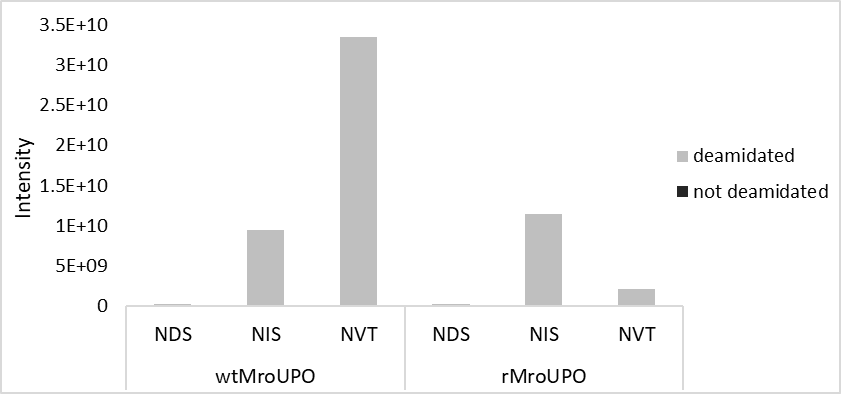

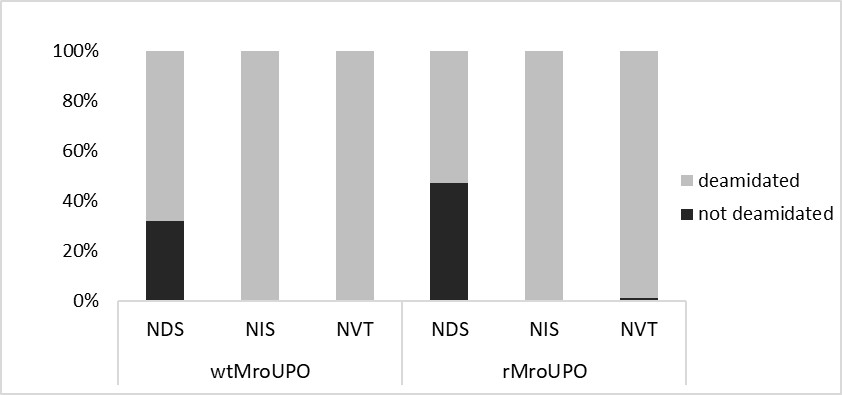


**A**

**B**


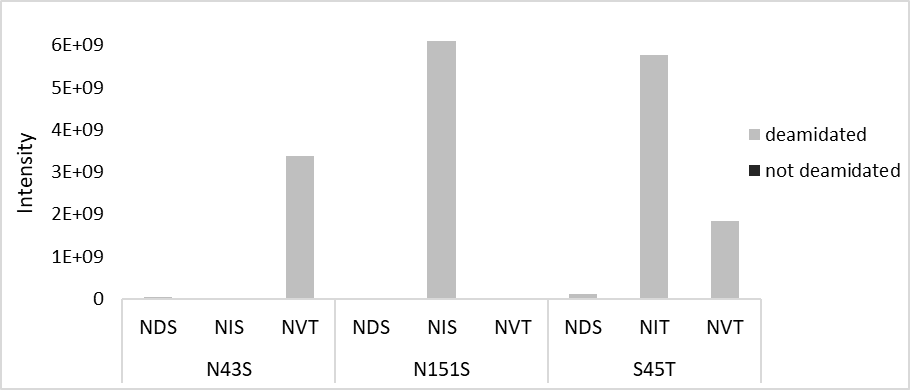

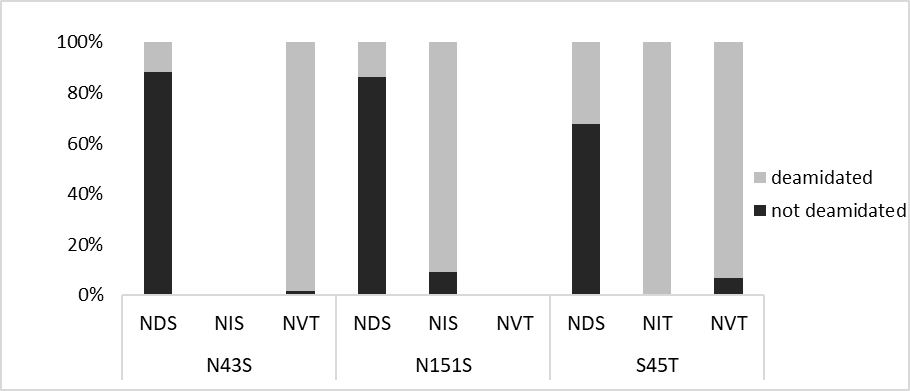


**C**

**D**

Figure S2: Analysis of the *N*-glycosylation status of different variants of *Mro*UPO. The proteins were treated with PNGase F, digested with trypsin and analyzed by mass spectrometry (MS). Asparagine residues in the potential sequons (N19-D-S; N43-I-S; N151-V-T) were analyzed to determine whether they are deamidated (indicating prior binding of *N*-acetylglucosamine) or not deamidated and therefore likely not glycosylated. Peptides with the respective sequons (deamidated or not deamidated) are shown as cumulated intensities for wt*Mro*UPO and r*Mro*UPO (A) and the variants r*Mro*UPO N43S; N151S; S45T (C) or as a percentage (B and D). Peptides with the potential sequon N130-I-S were not found in the MS data.

| Substrate | Kinetic constant | wt*Mro*UPO | r*Mro*UPO |
| --- | --- | --- | --- |
| DMP | K_m_ (mM) | 0.099 | 0.109 |
|  | k_cat_ (s^-1^) | 135.4 | 181.9 |
|  | k_cat_/K_m_ (mM^-1^ s^-1^) | 1,372 | 1,675 |
| NBD | K_m_ (mM) | 0.058 | 0.083 |
|  | k_cat_ (s^-1^) | 15.3 | 26.4 |
|  | k_cat_/K_m_ (mM^-1^ s^-1^) | 264 | 316 |
| VA | K_m_ (mM) | 0.266 | 0.357 |
|  | k_cat_ (s^-1^) | 47.9 | 63.2 |
|  | k_cat_/K_m_ (mM^-1^ s^-1^) | 180 | 177 |

Table S1: Kinetic parameters of wild-type and recombinant *Mro*UPO. Ultrafiltrated culture supernatants as well as purified r*Mro*UPO and wt*Mro*UPO were used for their determination. The data obtained are in the same order of magnitude.


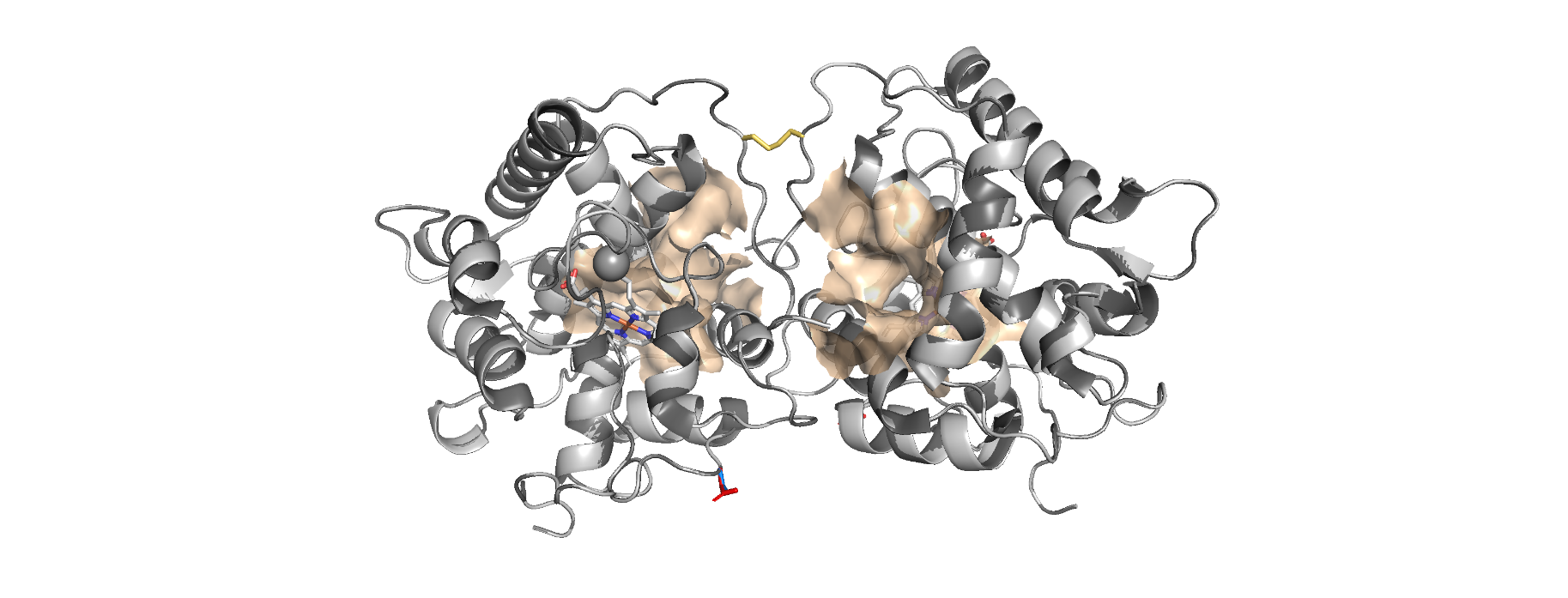

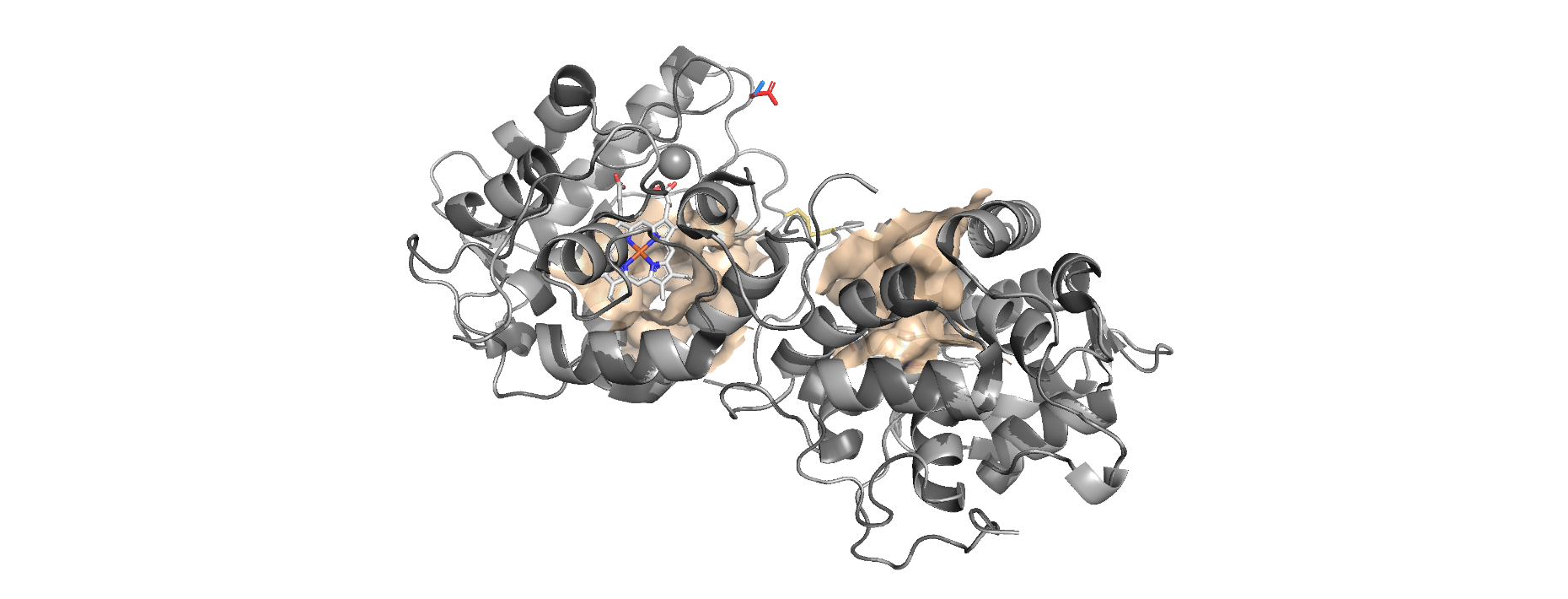

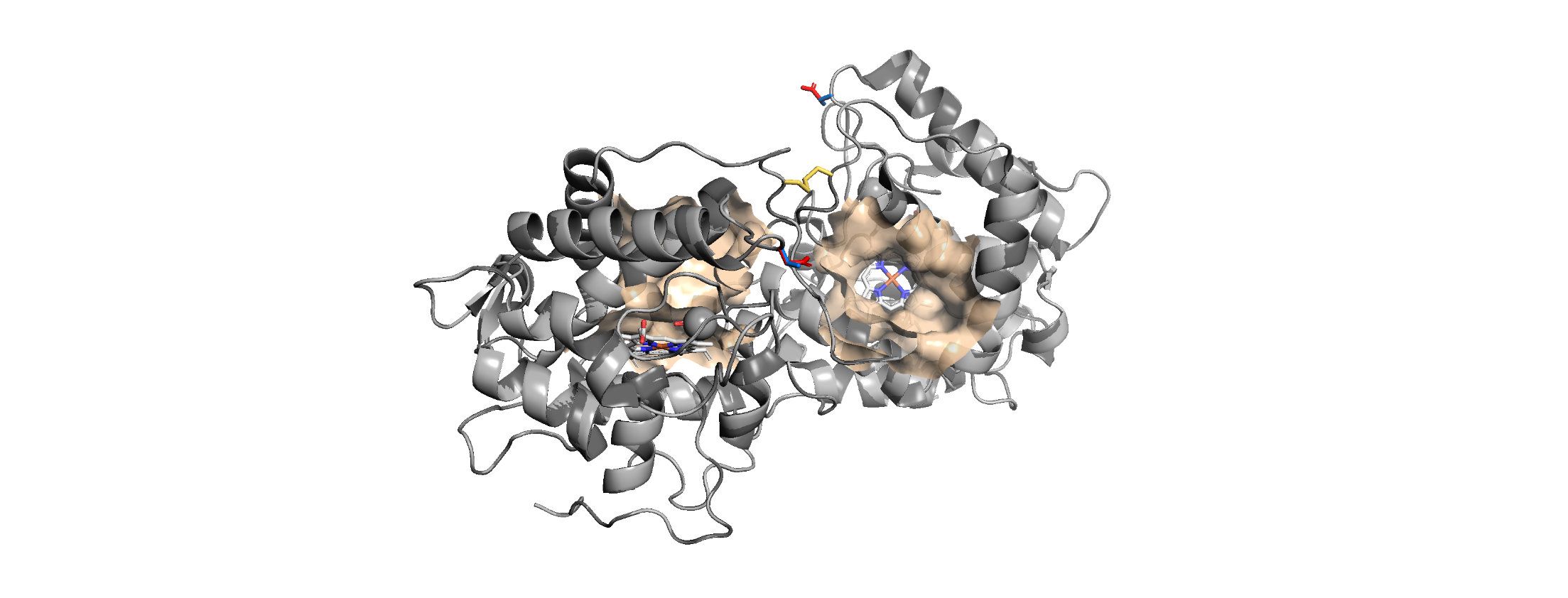


**B-1**

**A**

**B-2**


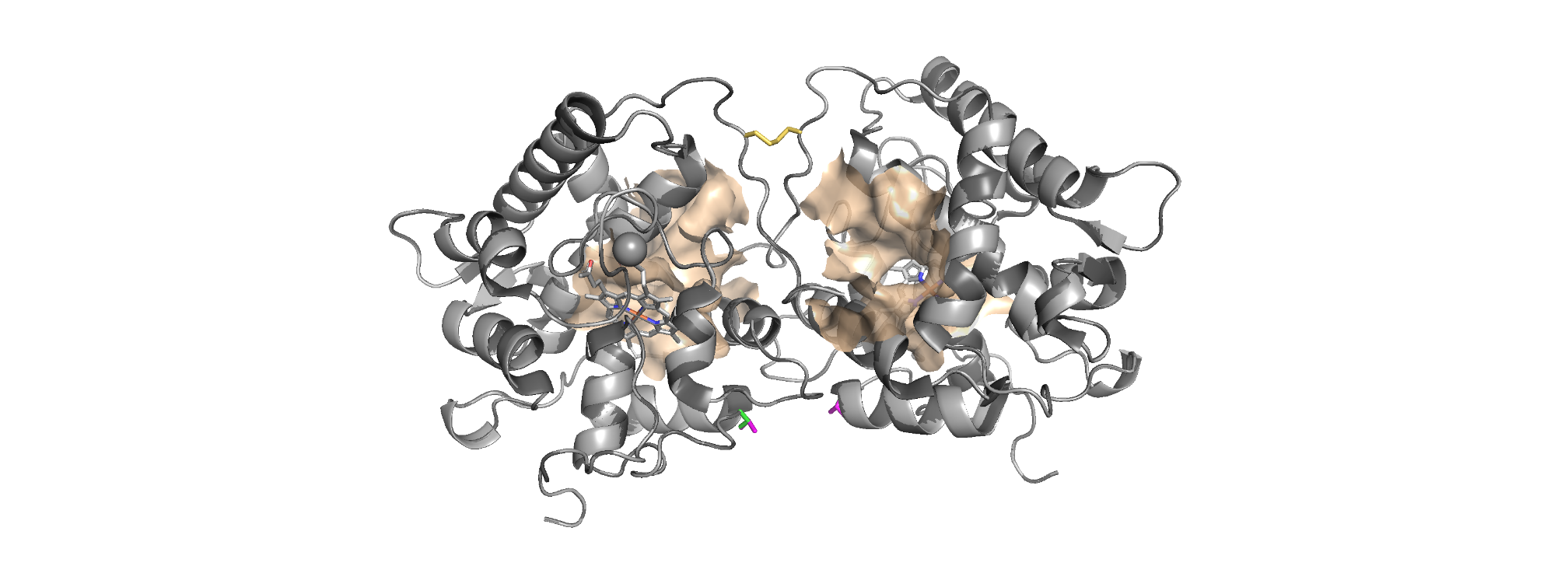


**C**

Figure S3: Superimpositions of models of recombinant *Mro*UPO variants (dark grey) and wild-type *Mro*UPO (light grey). The models were generated by homology modelling with PDB# 5FUJ as template using SWISS-MODEL and aligned with PDB# 5FUJ in PyMOL. Relevant asparagine residues that were exchanged in the variants N43S (A) and N151S (B-1 and B-2) are shown as red sticks, the inserted serine is shown as blue sticks. Figure S3 B-2 shows the dimer at a different angle. Serine (S45) in the wt*Mro*UPO (green stick) was replaced by threonine (magenta stick) in the variant S45T (C). **The RMSD between the aligned structures of** N43S and wt*Mro*UPO, N151S and wt*Mro*UPO and S45T and wt*Mro*UPO was 0.065 Å, 0.066 Å and 0.076 Å.

Protein sequence of *Mro*UPO

MKLAISSSLIALVSVTTALANSQDVVDFSAHPWKAPGPNDSRGPCPGLNTLANHGFLPRNGRNISVPMIVKAGFEGYNVQSDILILAGKIGMLTSREADTISLEDLKLHGTIEHDASLSREDVAIGDNLHFNEAIFTTLANSNPGADVYNISSAAQVQHDRLADSLARNPNVTNTDLTATIRSSESAFFLTVMSAGDPLRGEAPKKFVNVFFREERMPIKEGWKRSTTPITIPLLGPIIERITELSDWKPTGDNCGAIVLSPEL

*MroUPO* gene

ATGAAGTTGGCTATTTCTTCTTCCTTGATTGCCTTGGTTTCTGTTACTACTGCTTTGGCTAATTCCCAAGATGTTGTTGATTTTTCTGCTCATCCATGGAAAGCTCCAGGTCCAAATGATTCTAGAGGTCCATGTCCAGGTTTGAATACTTTAGCTAATCATGGTTTTTTGCCAAGAAACGGTAGAAACATCTCTGTTCCAATGATTGTCAAGGCTGGTTTTGAAGGTTACAACGTTCAATCCGATATTTTGATTTTGGCCGGTAAGATTGGTATGTTGACTTCTAGAGAAGCCGATACCATTTCCTTGGAAGATTTGAAATTGCACGGTACTATCGAACATGATGCCTCATTGTCAAGAGAAGATGTTGCTATTGGTGACAACTTGCATTTCAACGAAGCTATTTTCACTACCTTGGCTAACTCTAATCCAGGTGCTGATGTTTACAACATTTCTTCTGCTGCTCAAGTTCAACATGATAGATTGGCTGATTCTTTGGCTAGAAACCCAAATGTTACCAACACTGATTTGACCGCTACTATCAGATCTTCTGAATCTGCTTTCTTCTTGACCGTTATGTCTGCTGGTGATCCATTGAGAGGTGAAGCTCCAAAAAAGTTCGTTAACGTTTTCTTCAGAGAAGAAAGAATGCCAATCAAAGAAGGTTGGAAGAGATCTACTACCCCAATTACTATTCCATTATTGGGTCCAATCATCGAAAGAATCACCGAATTGTCTGATTGGAAACCTACTGGTGATAATTGTGGTGCTATAGTCTTGTCTCCAGAATTGTGA

**References**

Gröbe, G., Ullrich, R., Pecyna, M.J., Kapturska, D., Friedrich, S., Hofrichter, M., and Scheibner, K. (2011) High-yield production of aromatic peroxygenase by the agaric fungus Marasmius rotula. *AMB Express* **1**: 31.

Pinter, N., Glätzer, D., Fahrner, M., Fröhlich, K., Johnson, J., Grüning, B.A., et al. (2022) MaxQuant and MSstats in Galaxy Enable Reproducible Cloud-Based Analysis of Quantitative Proteomics Experiments for Everyone. *J Proteome Res* **21**: 1558–1565.

Theorell, H., Maehly, A.C., Dam, H., and Kinell, P.-O. (1950) Untersuchungen an künstlichen Peroxydasen. *Acta Chem Scand* **4**: 422–434.

Waterhouse, A., Bertoni, M., Bienert, S., Studer, G., Tauriello, G., Gumienny, R., et al. (2018) SWISS-MODEL: homology modelling of protein structures and complexes. *Nucleic Acids Research* **46**: W296–W303.
